# What Comes First, Job Burnout or Secondary Traumatic Stress? Findings from Two Longitudinal Studies from the U.S. and Poland

**DOI:** 10.1371/journal.pone.0136730

**Published:** 2015-08-25

**Authors:** Kotaro Shoji, Magdalena Lesnierowska, Ewelina Smoktunowicz, Judith Bock, Aleksandra Luszczynska, Charles C. Benight, Roman Cieslak

**Affiliations:** 1 Trauma, Health, and Hazards Center, University of Colorado Colorado Springs, Colorado Springs, Colorado, United States of America; 2 Department of Psychology, University of Social Sciences and Humanities, Warsaw, Poland; 3 Department of Psychology, University of Social Sciences and Humanities, Wroclaw, Poland; 4 Department of Psychology, University of Colorado Colorado Springs, Colorado Springs, Colorado, United States of America; University of Toledo, UNITED STATES

## Abstract

This longitudinal research examined the directions of the relationships between job burnout and secondary traumatic stress (STS) among human services workers. In particular, using cross-lagged panel design, we investigated whether job burnout predicts STS at 6-month follow up or whether the level of STS symptoms explains job burnout at 6-month follow-up. Participants in Study 1 were behavioral or mental healthcare providers (*N* = 135) working with U.S. military personnel suffering from trauma. Participants in Study 2 were healthcare providers, social workers, and other human services professions (*N* = 194) providing various types of services for civilian trauma survivors in Poland. The cross-lagged analyses showed consistent results for both longitudinal studies; job burnout measured at Time 1 led to STS at Time 2, but STS assessed at Time 1 did not lead to job burnout at Time 2. These results contribute to a discussion on the origins of STS and job burnout among human services personnel working in highly demanding context of work-related secondary exposure to traumatic events and confirm that job burnout contributes to the development of STS.

## Introduction

Job burnout and secondary traumatic stress (STS) have been recognized as the crucial consequences of extreme job demands in human services professionals [1,2]. Such demands may include frequent and intense contact with traumatized clients and chronic exposure to traumatic content at work [1,2]. Job burnout generally refers to a response to a broad range of occupational stressors and chronic tediousness in the workplace, and it is often characterized by symptoms such as emotional exhaustion, depersonalization, or a lack of personal accomplishment [3]. In turn, STS may be defined as a psychological response to very specific type of stressor in work environment, namely an indirect (secondary) exposure to traumatic contents in professional contacts with traumatic stress survivors [2]. STS may be manifested by symptoms similar to posttraumatic stress disorder [2].

High prevalence of job burnout was demonstrated across various occupational groups, reaching up to 67% for burnout in the community of mental health workers [4]. The prevalence of STS was found to vary from 15.2% among social workers [5] to 19.2% among U.S. mental health providers working in military [6], and up to 39% among juvenile justice education workers [7]. Results of a recent meta-analysis by Cieslak and colleagues [8] indicated strong associations between job burnout and STS among human services professionals. Unfortunately, the vast majority of studies investigating this relationship is of cross-sectional design; therefore, there is no evidence clarifying whether job burnout leads to STS, STS leads to job burnout, or this relationship is bi-directional (i.e., whatever develops first, increases the likelihood of developing another type of consequences). Establishing what comes first in the job burnout-STS relationship could be an essential step guiding prevention, treatment, and education programs for human services professionals, enabling them to reduce negative consequences of work stress. To fill this void, our two longitudinal studies investigated the directions of the relationships between job burnout and STS.

### Job Burnout

Although there are many conceptualizations of job burnout [9], the three-factor model [3] and the two-factor model [10] have been most commonly used. Job burnout has been traditionally conceptualized as encompassing three dimensions, emotional exhaustion, depersonalization, and a lack of personal accomplishment [3]. However, several work-related stressors and outcomes are more strongly correlated with emotional exhaustion and depersonalization than with a lack of personal accomplishment [11], whereas a lack of personal accomplishment forms strong associations with personal resources, e.g. self-efficacy [12]. In a response to findings showing a distinct function of a lack of personal accomplishment compared to other two burnout components, Demerouti et al. [10] proposed the two-factor model of job burnout, accounting for exhaustion and disengagement components. Exhaustion refers to the affective, physical, and cognitive states whereas the disengagement refers to distancing oneself from the entire spectrum of work-related aspects, e.g., job tasks, co-workers, work in general [10]. The present study examines the components that are strongly associated with work-related stressors; therefore, we used the two-factor model.

Research has consistently shown the associations between job burnout and work-related factors. Meta-analytical studies found significant relationships between job burnout and risk factors such as high job demands (e.g., workload, role conflict) or low job resources (e.g., control, autonomy at work) [11,13]. Another important predictor for job burnout is years of work experience [14–16]. Whereas the effects of many burnout determinants, such as job ambiguity or supervisor support, may vary across cultures [17], the links between years of work experience and work-related outcomes may be similar across the cultures [18]. Therefore, our research conducted in two cultures accounted for the predictor which may operate similarly across the countries, that is years of work experience.

### Secondary Traumatic Stress

Consequences of work-related indirect exposure to traumatic events have been conceptualized using several terms which have been used interchangeably. The constructs which are used most often include vicarious traumatization [19], compassion fatigue [2], and secondary posttraumatic stress disorder, also called secondary traumatic stress (STS) [20]. These constructs are overlapping, but they are not identical in content or theoretical foundations. STS accounts for three clusters of symptoms, such as intrusion, re-experiencing, and avoidance [20], whereas vicarious traumatization has core elements such as the professional’s engagement at work and cognitive effects of indirect exposure to traumatic events [19]. In turn, compassion fatigue involves any emotional duress and burnout components [2]. In sum, the theoretical framework proposed by Bride et al. [20], captures STS as a construct, which is clearly distinct from job burnout, and therefore, the present research focuses on STS.

Indirect exposure to trauma is a necessary condition for developing STS. There are a number of indices measuring indirect exposure to traumatic events, including diversity, volume, ratio, and frequency [6]. Across these exposure indices, the frequency of indirect exposure has been identified as the most consistent determinant of STS [20–22]. Therefore, we included the frequency of indirect exposure as the determinant of STS.

Due to the high frequency of indirect exposure to traumatic events, some human services professionals are particularly at risk of developing STS. Professions that have been found to generate high risk of developing STS include social workers [5], child protective service workers [23], military health providers [6], and general trauma therapists [1]. Military behavioral healthcare providers, social workers, and trauma therapists were recruited to participate in our research.

Research on secondary traumatic stress faces some conceptual challenges. For example, McNally [24] argued that PTSD-like symptoms due to indirect exposure to traumatic materials may be an example of conceptual ‘bracket creep’ and that creating the construct of STS may be motivated by making treatment for PTSD-like symptoms reimbursable. This issue may be of particular relevance in cases when the diagnosis and subsequent reimbursed treatment of STS is considered. Importantly, the critique of STS concept is particularly strong in the context of people exposed to traumatic material via mass media [25]. However, DSM-V [26] clearly excluded exposure via electronic or printed media as a condition to develop traumatic stress disorder. Furthermore, if the aim of study is not to diagnose but to identify the determinants and consequences of the intensity or frequency of STS symptoms, the ‘bracket creep’ argument becomes weaker. Finally, there is no doubt that some extremely shocking, horrifying, and gruesome traumatic materials may be brought indirectly to professionals working with refugees [27], survivors of terrorist attacks [28], or military personnel [6]. Human services professionals may develop PTSD-like symptoms from repeated hearing and sharing in the details of the stories of survivors (for meta-analysis see 7). Using the concept of STS to professionals vicariously exposed to traumatic events may serve to de-stigmatize the reactions of first responders and reinforce the need for training and preventive care [29]. In sum, regardless the controversies, there are strong arguments for considering STS as one of key issues in well-being of human services professionals exposed to trauma through their work [6].

### Associations between Job Burnout and Secondary Traumatic Stress

Theoretical frameworks, addressing consequences of work-related stress among human services professionals indirectly exposed to trauma, assume that job burnout and STS may co-occur [30]. However, they do not provide suggestions about the uni- or bi-directional character of these relationships [30]. In contrast, more general stress frameworks such as conservation of resources theory (COR) [31] may provide arguments for a uni-directional relationship between job burnout and STS.

According to COR, the exposure to a broad range of stressors may deplete a broad range of resources and lead to resource exhaustion [31]. Work-related stressors are the examples of such broad range of stressors. This broad range of stressors may consequently lead to exhaustion of a broad range of resources. Emotional exhaustion, which is a component of job burnout, may represent one of the facets of loss/exhaustion of resources. COR [31] suggests that a loss of a broad range of resources (including emotional exhaustion and reduced motivation to engage in various challenging tasks) may in turn increase the likelihood of developing specific negative consequences after exposure to subsequent specific stressors [31]. STS and indirect exposure may represent such specific consequences and stressors, respectively.

An indirect exposure to traumatic stress may lead to the depletion of a relatively limited amount of resources (compared to a loss of resources following a broad range of work-related stressors). After a secondary exposure to a traumatic event, the availability of some resources should remain unaffected (e.g. organizational support, autonomy). Therefore, secondary exposure and its consequences such as STS may have relatively small effects on subsequent burnout.

To the best of our knowledge, there have been no longitudinal studies evaluating two competing models, assuming that burnout leads to STS or that STS leads to burnout. Van der Ploeg and Kleber [32] tested only one of the two competing models and found that symptoms of post-traumatic stress at Time 1 might lead to emotional exhaustion at Time 2. Another longitudinal study indicated that job burnout at Time 1 led to depression at Time 2 [33]. Overall, cross-sectional studies indicated a strong association between job burnout and STS [8]. As experimental studies inducing either job burnout or STS are not an option from obvious ethical reasons, longitudinal studies analyzing the two competing models could bring us closer to answer the question of the directions in job burnout-STS associations. Importantly, a recent meta-analysis [8] indicated that the job burnout-STS associations are moderated by the country and the language in which the measurement is taken. Therefore, investigation of the directions in the job burnout-STS associations needs to be conducted across cultures.

### Aims of Present Studies

We tested the directions of the associations between job burnout and STS. The investigation was conducted in two independent samples of human services professionals, exposed to work stress and indirectly exposed (through their clients) to traumatic events. In particular, we explored three alternative hypotheses:

1. Job burnout at Time 1 would predict STS at Time 2 whereas STS at Time 1 would not predict job burnout at Time 2.
2. STS at Time 1 would predict job burnout at Time 2 whereas job burnout at Time 1 would not explain STS at Time 2.
3. Job burnout at Time 1 would explain STS at Time 2, and STS at Time 1 would predict job burnout at Time 2.

The three hypotheses were tested in a longitudinal study enrolling the U.S. behavioral healthcare providers working with military personnel suffering from trauma. Next, the findings were replicated in a sample of Polish human services professionals working with civilians exposed to various traumatic experiences.

## Study 1

### Method

#### Participants

In Study 1, data were collected among behavioral and mental healthcare providers, working with the U.S. military personnel. Professionals meeting the following criteria were included: (a) working at least one year as a behavioral healthcare provider, clinical psychologist, counselor, or social worker; (b) providing services for military personnel; and (c) experiencing indirect exposure to traumatic stress through their work.

In total, 294 providers met the inclusion criteria and completed the online survey at Time 1 (T1). Participants’ mean age was 48.87 (*SD* = 12.76). Table 1 displays further demographic information. Six months later (Time 2; T2), 135 professionals (mean age = 50.62 years old [*SD* = 12.58]) completed the follow-up measurement. Among those who provided their data at T2, there were 50 clinical psychologists (37.0%), 39 counselors (28.9%), 29 social workers (20.7%), and 9 healthcare providers (6.7%). Respondents had been exposed to indirect traumatic events such as life threatening illness or injury (91.9%), military combat (91.1%), sudden unexpected death of someone close (90.4%), sexual assault (87.4%), physical assault (85.9%), transportation accidents (83.7%), natural disasters (68.9%), and life threatening crime (57.0%).

**Table 1:** Descriptive Statistics for Demographics for Study 1 (U.S. Data) and Study 2 (Polish Data).

Measure	Levels	Study 1		Study 2	
		Time 1	Time 2	Time 1	Time 2
**Gender**					
	Female	66.3% (195)	71.1% (96)	76.3% (232)	79.9% (155)
	Male	33.7% (99)	28.9% (39)	22.7% (69)	18.6% (36)
**Relationship status**					
	In long-term relationship	76.2% (224)	72.6% (98)	73.7% (224)	77.3% (150)
	Not in long-term relationship	21.4% (63)	25.2% (34)	25.7% (78)	22.2% (43)
**Highest degree**					
	High school	0.3% (1)	0 (0%)	20.4% (62)	18.0% (35)
	Associate’s degree	0.3% (1)	0 (0%)	-	-
	Bachelor’s degree	2.0% (6)	1.5% (2)	21.4% (65)	19.1% (37)
	Master’s degree	45.2% (133)	51.1% (69)	56.6% (172)	61.3% (119)
	Doctorate degree	52.0% (153)	47.4% (64)	1.0% (3)	0.5% (1)

*Note*. Sample size for Study 1 at T1 = 294. Sample size for Study 1 at T2 = 135. Sample size for Study 2 at T1 = 304. Sample size for Study 2 at T2 = 194. Some percentages did not add up to 100% because of missing data. Long-term relationship included married couples and couples in a committed relationship.

#### Measurements

Participants completed a set of questionnaires assessing job burnout, STS, and demographic information at T1 and T2.

Job burnout: The Oldenburg Burnout Inventory (OLBI) [34] is a 16-item questionnaire used to assess exhaustion (eight items) and disengagement (eight items). Respondents rate the degree of agreement for each item on a 5-point scale ranging from 1 (*strongly disagree*) to 5 (*strongly agree*). Sample items included “During my work, I often feel emotionally drained” and “Lately, I tend to think less at work and do my job almost mechanically.” Cronbach’s alpha coefficients were .81 at T1 and .85 at T2 for the exhaustion subscale and .85 at T1 and .86 at T2 for the disengagement subscale.

Secondary traumatic stress: The Secondary Traumatic Stress Scale (STSS) [20] is a 17-item measure of the frequency of STS symptoms in the previous month. Responses are provided on a 5-point scale ranging 1 (*never*) to 5 (*very often*). Sample items included “It seems as if I was reliving the trauma(s) experienced by my patient(s)”, “I had little interest being around others”, and “I felt jumpy.” Cronbach’s alpha was .93 for both T1 and T2.

Indirect exposure to trauma: The Secondary Trauma Exposure Scale [6] is a list of 10 events. It was designed to measure indirect exposure to traumatic stress among behavioral healthcare providers. Participants indicate whether they have experienced each event (e.g., natural disaster, sexual assault, military combat, exposure to a war-zone) through their clients. The frequency of indirect exposure was measured by referring the list of events with one item on a 7-point scale ranging between 1 (*never*) to 7 (*every day*).

Demographics: Participants completed background questions such as gender, age, work experience in years, education, type of profession, and relationship status.

#### Procedures

The Institutional Review Board at the University of Colorado Colorado Springs approved this study. The recruitment procedures were described elsewhere [6,35,36]. Participants were asked to indicate that they agreed to participate on the online informed consent form before they started answering the survey. Six months after completion of the T1 survey, professionals who agreed to participate in the T2 survey received an email invitation and a link to the T2 online survey. The mean time between the T1 and T2 was 195.80 days (*SD* = 20.00).

#### Analytical strategies

**Fig 1:**
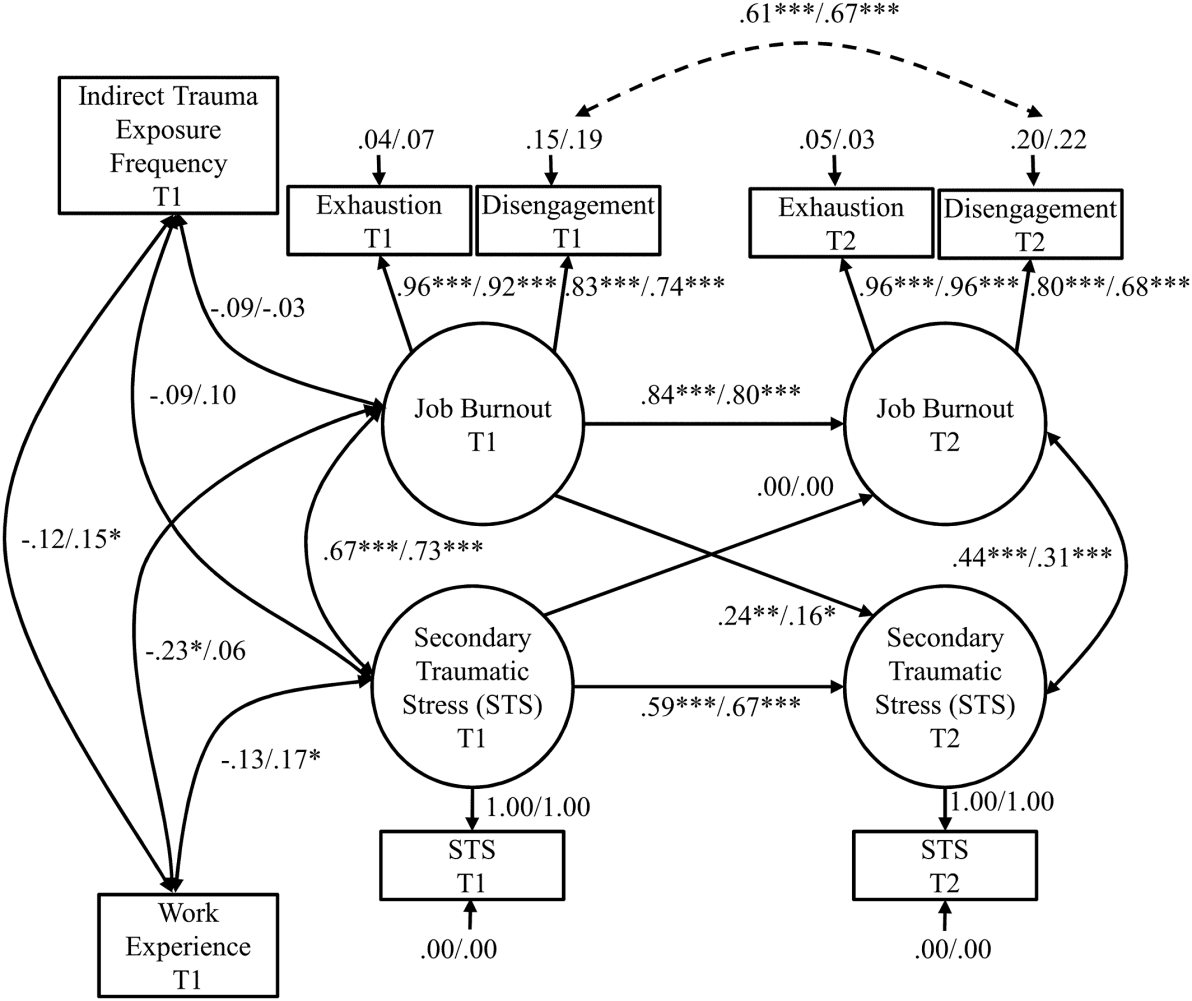
Standardized Coefficients in the Cross-Lagged Panel Analysis for the Model Examining the Directionality between Job Burnout and STS. The covariation between the error terms for disengagement at Time 1 and disengagement at Time 2 (dotted line) was added based on the modification indices. This model represents the final model with the coefficient for the relationship between STS at Time 1 and job burnout at Time 2 constrained to zero. The coefficients for the relationship between exhaustion and the job burnout latent variable were constrained to one. Values before the slash indicate values for the Study 1, and those after the slash indicate the values for the Study 2. T1 = Time 1; T2 = Time 2. *** *p* < .001; ** *p* < .01; * *p* < .05.

To test the relationships between job burnout and STS, a cross-lagged panel analysis was conducted using structural equation modeling with AMOS version 22 (IBM). The hypothesized model included cross-lagged associations between job burnout at T1 and STS at T2 and between STS at T1 and job burnout at T2 (see Fig 1). The T1 indicators of job burnout and STS were assumed to covary. The T2 indicators of job burnout and STS were also assumed to covary. Work experience (T1) and the frequency of exposure to indirect traumatic stress (T1) were included as the covariates of both STS (T1) and job burnout (T1). The latent variables representing job burnout at both measurement points were loaded by two observed variables, exhaustion and disengagement (Fig 1). The latent variables representing STS at T1 and T2 were loaded by one observed variable, STS, measured at T1 or T2, respectively.

The hypothesized model was estimated using maximum likelihood procedure. Assumptions for univariate and multivariate normality were met. The following indices were used to evaluate the model-data fit: Root mean square error of approximation (RMSEA), cutoff < .10 [37]; comparative fit index (CFI), cutoff > .90 [38]; Tucker Lewis Index (TLI), cutoff > .90 [38]; and standardized root mean residual (SRMR), cutoff < .08 [38].

To test the directions of the relationships between job burnout and STS, the hypothesized model was compared with two nested models. In the first nested model the path representing the effect of STS (T1) on job burnout (T2) was constrained to zero. In the second nested model, the path representing the effect of job burnout (T1) on STS (T2) was constrained to zero.

All analyses were conducted in the sample of completers (*N* = 135). Missing data were replaced using imputation with the maximum likelihood estimation method [39,40]. Items of exhaustion at T1 and T2, disengagement at T1 and T2, STS at T1 and T2, the frequency of indirect trauma exposure, and work experience were included in the imputation. Little’s test [41] showed that data were missing completely at random (MCAR) for items of STS at T1, χ^2^(9) = 7.46, *p* = .59, STS at T2, χ^2^(53) = 54.48, *p* = .42, exhaustion at T1, χ^2^(35) = 30.29, *p* = .70, disengagement at T1, χ^2^(7) = 9.17, *p* = .24, and disengagement at T2, χ^2^(11) = 8.27, *p* = .69. However, items of exhaustion at T2 were not MCAR, χ^2^(32) = 57.08, *p* = .01. Because only 0.74% of all data for exhaustion were missing at T2, we used the maximum likelihood estimation imputation for these items as well. In total, 0.49% of data were missing and imputed.

### Results

#### Preliminary Analyses

To examine whether the constructs of job burnout and STS were measured in a sufficiently distinctive way, Pearson’s correlations for the items of the OLBI and the items of the STSS were calculated (*r* range: .05 to .43). The highest correlation between a job burnout item and an STS item was .43 (18.8% of shared variance), indicating that the two concepts were distinct (see a correlation matrix in S1 Table).

Table 2 displays means, standard deviations, and Pearson’s correlation coefficients for all study variables. Attrition analysis showed no significant differences between completers and dropouts in disengagement at T1, *t*(292) = 0.82, *p* = .41, exhaustion at T1, *t*(292) = 0.90, *p* = .37, STS at T1, *t*(292) = 0.14, *p* = .89, age, *t*(288) = 0.08, *p* = .94, gender, χ^2^(1) = 0.40, *p* = .53, profession, χ^2^(3) = 0.28, *p* = .96, relationship status, χ^2^(1) = 0.84, *p* = .36, and education, χ^2^(4) = 4.89, *p* = .30. All STS and job burnout indicators were correlated (*r* ranged from .48 to .80, all *p*s < .001).

**Table 2:** Means, Standard Deviations, Pearson’s Correlations among Study Variables for Study 1 (below Diagonal) and Study 2 (above Diagonal).

Measure									Mean (*SD*)		
	1	2	3	4	5	6	7	8	Study 1	Study 2	*t*
1. Emotional exhaustion at T1	-	.69***	.68***	.49***	.68***	.60***	.04	-.02	2.54 (0.70)	2.82 (0.68)	3.61***
2. Emotional exhaustion at T2	.77***	-	.58***	.66***	.57***	.62***	.09	.01	2.53 (0.76)	2.80 (0.60)	3.45***
3. Depersonalization at T1	.80***	.64***	-	.74***	.52***	.45***	.02	-.07	2.35 (0.70)	2.71 (0.64)	4.75***
4. Depersonalization at T2	.67***	.76***	.77***	-	.42***	.42***	.00	-.00	2.40 (0.76)	2.77 (0.65)	4.61***
5. STS at T1	.64***	.57***	.54***	.48***	-	.79***	.17*	.10	1.88 (0.61)	2.33 (0.68)	6.28***
6. STS at T2	.59***	.67***	.52***	.55***	.75***	-	.23**	.14	1.76 (0.62)	2.28 (0.69)	7.14***
7. Work experience in years at T1	-.09	-.03	-.10	-.10	-.10	.04	-	.15*	15.70 (10.38)	10.38 (8.52)	5.09***
8. Indirect trauma frequency at T1	-.19*	-.24**	-.31***	-.29***	-.13	-.18*	-.11	-	6.16 (1.12)	4.79 (1.74)	8.06***

*Note*. Correlations in lower diagonal region show values for U.S. data (Study 1). Correlations in upper diagonal region show values for Polish data (Study 2). Sample size for Study 1: *N* = 135. Sample size for Study 2: *N* = 194. STS = secondary traumatic stress; T1 = Time 1; T2 = Time 2.

*p < .05

**p < .01

***p < .001. *t*-tests are conducted for each variable between Study 1 and Study 2.

#### Results of Cross-Lagged Panel Analysis

To test the associations between job burnout and STS, a cross-lagged panel analysis was performed (see Fig 1). The analysis indicated that the data did not fit the hypothesized model very well with RMSEA = .169, CFI = .922, TLI = .831, and SRMR = .041. Based on the modification indices, we modified the hypothesized model by covarying error variances for disengagement at T1 and T2. Results for the modified hypothesized model showed acceptable model fit, RMSEA = .074, CFI = .986, TLI = .968, and SRMR = .041. This modified hypothesized model was used for further analysis and model comparisons. The results suggested that relationship between job burnout at T1 and STS at T2 was significant, whereas the relationship between STS at T1 and job burnout at T2 was not significant.

To further test the direction of the associations between job burnout and STS, the modified hypothesized model was compared with two nested models (see Table 3). The difference between the modified hypothesized model and the first nested model (with the path representing the effect of T1 STS on T2 job burnout constrained to zero) was not significant. In contrast, the difference between the modified hypothesized model and the second nested model (with the path representing the effect of T1 job burnout on T2 STS constrained to zero) was significant. Therefore the second nested model should be rejected. Based on the results of the cross-lagged panel analysis, the first nested model with the relationship between STS at T1 and job burnout at T2 constrained to zero should be accepted as a final model.

**Table 3:** Goodness-Of-Fit Statistics for Comparisons Between the Modified Hypothesized and the Nested Models in Two Studies.

Study	Model Description	χ^2^	χ^2^/*df*	NFI	Δχ^2^	ΔNFI
**Study 1**						
	The modified hypothesized model	20.90	1.74	.969	-	-
	First nested model: The path from STS (T1) to job burnout (T2) constrained to zero	21.77	1.68	.968	0.88	.001
	Second nested model: The path from job burnout (T1) to STS (T2) constrained to zero	28.09	2.16	.959	7.19**	.011
**Study 2**						
	The modified hypothesized model	13.70	1.14	.984	-	-
	First nested model: The path from STS (T1) to job burnout (T2) constrained to zero	14.43	1.11	.983	0.74	.001
	Second nested model: The path from job burnout (T1) to STS (T2) constrained to zero	17.69	1.36	.979	3.99*	.005

*Note*. The Δχ^2^ indicates a change in a χ^2^ from the modified hypothesized model. A significant Δ χ^2^ value indicates that the model was significantly different from the modified hypothesized model. STS = secondary traumatic stress; T1 = Time 1; T2 = Time 2.

**p < .01

*p < .05.

### Discussion

Results of Study 1 provided support for the first hypothesis and indicated that the cross-lagged pathway from job burnout at T1 to STS at T2 represents the essential and significant link between the two variables. High job burnout at T1 predicted higher STS measured six months later. These results need to be cross-validated and replicated in the context of different professions, culture, and organizations. In Study 2, we recruited employees indirectly exposed to trauma, working in cultural and demographic contexts different from those in Study 1.

## Study 2

### Method

#### Participants

In Study 2, Polish healthcare and social workers providing services for civilians who had experienced traumatic events were recruited. Inclusion criteria were: (a) working for at least one year as a healthcare provider, social worker, or first responder; (b) providing services for civilians exposed to traumatic events; and (c) experiencing indirect exposure to trauma at work.

Three hundred and four professionals (mean age = 35.27 years old [*SD* = 8.43]) met the inclusion criteria and completed the online survey at Time 1 (T1). Table 1 displays demographic information. Of those who completed the T1 survey, 194 participants (mean age = 35.10 years old [*SD* = 8.08]) provided their data six months later (Time 2; T2). The T2 sample consisted of 87 healthcare providers (44.8%), 81 social workers (41.8%), and 23 other professions (11.9%). All participants were indirectly exposed to different types of traumatic events at work, such as life-threatening injury or illness (88.1%), physical assault (87.1%), sudden unexpected death of someone close (83.5%), transportation accidents (71.1%), sexual assault (50.5%), and natural disasters (30.4%). Only 7.2% of participants were indirectly exposed to combat-related traumatic events.

#### Measurements

Participants completed the same set of measures as in Study 1. All measures had acceptable reliability: for exhaustion we obtained αs of .82 (T1) and .78 (T2); for disengagement αs of .79 (T1) and .81 (T2), and for STS αs of .92 (T1) and .93 (T2). As in Study 1, instructions for all instruments were modified; participants were asked to provide their responses in the context of work-related indirect exposure to traumatic events. Back-translation procedures were applied to develop the Polish versions of the questionnaires.

#### Procedures

The Institutional Review Board at the University of Social Sciences and Humanities, Warsaw, Poland approved the study. Data were collected using an online survey. Before participants started answering the online survey, they were asked to indicate whether they agreed to participate on the online informed consent form. The recruitment procedures were described elsewhere [35,36]. The mean time elapsed between the T1 and T2 was 162.35 days (*SD* = 39.51).

#### Analytical Strategies

To test the relationships between job burnout and STS, we performed a longitudinal cross-lagged panel analysis using the same procedure and software as in Study 1 (see Analytical Procedures in Study 1). Assumptions of univariate and multivariate normality for structural equation modeling were met.

In addition, to examine the consistency of the factor structure of the OLBI and the STSS in the U.S. sample (T1: *N* = 294) and the Polish sample (T1: *N* = 304), tests for measurement invariance were conducted using a series of confirmatory factor analysis, following the suggestions by Prince [42]. For the test of invariance of the OLBI, eight items for the emotional exhaustion subscale were assumed to load on the first latent variable, and eight items for the depersonalization subscale were assumed to load on the second latent variable. The two latent variables, representing emotional exhaustion and depersonalization, were assumed to covary. In the test of invariance of the STSS, five items from the intrusion subscale were assumed to load on the first latent variable, seven items from the avoidance subscale were assumed to load on the second latent variable, and five items from the arousal subscale were assumed to load on the third latent variable. The latent variables for these subscales were assumed to covary. Such hypothesized models were then compared with nested model assuming that factor loadings, variances, and structural covariances are equal across the two samples.

All analyses were conducted in the sample of completers (*N* = 194). As in Study 1, missing data were replaced using imputation with the maximum likelihood estimation method. With gender and profession as references, the Little’s test [41] showed that data were missing completely at random for items of STS at T1, χ^2^(177) = 191.08, *p* = .22, STS at T2, χ^2^(188) = 201.88, *p* = .23, exhaustion at T1, χ^2^(28) = 39.91, *p* = .07, exhaustion at T2, χ^2^(25) = 30.07, *p* = .22, disengagement at T1, χ^2^(21) = 17.01, *p* = .71, and disengagement at T2, χ^2^(39) = 33.62, *p* = .71. In total, 0.81% of the values were imputed.

### Results

#### Preliminary Analyses

Pearson’s correlations among the OLBI items and the STSS items (*r* range:-.14 to .51) indicated that the highest correlation was .51 (25.8% of shared variance), suggesting that job burnout and STS are two distinct concepts (see a correlation matrix in S2 Table). Table 2 displays means, standard deviations, and Pearson’s correlation coefficients for variables in Study 2.

Attrition analysis showed no significant differences between completers and dropouts in disengagement at T1, *t*(302) = 1.22, *p* = .22; exhaustion at T1, *t*(302) = 0.09, *p* = .93, STS at T1, *t*(302) = 0.59, *p* = .55, age, *t*(275) = 0.65, *p* = .52, profession, χ^2^(2) = 2.49, *p* = .29, intimate relationship status, χ^2^(1) = 3.24, *p* = .07, and education, χ^2^(3) = 5.63, *p* = .13. However, there were more women among completers than among dropouts, χ^2^(1) = 4.61, *p* = .03. STS and job burnout indicators in Study 2 were significantly correlated, with *r* ranging from .42 to .79, all *p*s < .001. The levels of STS and job burnout at T1 and T2 were higher in Study 2 than the respective values obtained in Study 1 (see Table 2).

The test of measurement invariance for the OLBI between the U.S. sample and the Polish sample showed that good model-data fit for the hypothesized unconstrained model (with assumed 10 covariance between error variances), RMSEA = .050, CFI = .924, TLI = .900, and SRMR = .049. The hypothesized model without any constrains was significantly different from the nested model with factor loadings, variances, and structural covariances constrained to be equal, Δχ^2^ = 119.80, *p* < .001, ΔNFI = .031. Additionally, the hypothesized model was significantly different from the nested model with variances constrained to be equal, Δχ^2^ = 93.04, *p* < .001, ΔNFI = .024, and the nested model with the structural covariances constrained to be equal, Δχ^2^ = 14.70, *p* < .01, ΔNFI = .004. However, the hypothesized model was not significantly different from the model with factor loadings constrained to be equal, Δχ^2^ = 19.76, *p* = .14, ΔNFI = .005. Therefore, the nested model with factor loadings constrained to be equal was accepted, indicating that factor loadings of the OLBI were consistent across the two samples.

Results of the test of measurement invariance for the STSS between the U.S. sample and the Polish sample showed good model-data fit for hypothesized unconstrained model (with assumed 10 covariance between error variances), RMSEA = .056, CFI = .922, TLI = .900, and SRMR = .039. The hypothesized model without constrains was significantly different from the nested model with factor loadings, variances, and structural covariances constrained to be equal, Δχ^2^ = 297.28, *p* < .001, ΔNFI = .054, the nested model with factor loadings constrained to be equal, Δχ^2^ = 112.69, *p* < .001, ΔNFI = .021, the nested model with variances constrained to be equal, Δχ^2^ = 166.99, *p* < .001, ΔNFI = .031, and the nested model with structural covariances constrained to be equal, Δχ^2^ = 23.88, *p* < .001, ΔNFI = .004. Thus, none of the nested models was accepted for the STSS. Qualitative inspection of factor coefficients showed that one item (“I felt emotionally numb”) positively loaded on the avoidance latent variable in the U.S. sample whereas the same item nonsignificantly loaded on the avoidance latent variable, which might have contributed the difference in the factor structure of the STSS across the samples.

#### Results of Cross-Lagged Panel Analysis

The cross-lagged panel analysis conducted for the hypothesized model (without the covariance between error terms for disengagement at T1 and T2) indicated poor model-data fit, RMSEA = .190, CFI = .887, TLI = .758, and SRMR = .053. As in Study 1, the hypothesized model was modified; the error terms for disengagement at T1 and T2 were assumed to covary. The results obtained for the modified hypothesized model yielded good model-data fit, RMSEA = .027, CFI = .998, TLI = .995, and SRMR = .026. This modified hypothesized model was used in further comparisons with the nested models.

As in Study 1, to test the direction of the associations between job burnout and STS the modified hypothesized model was compared with two nested models (see Table 3). The difference between the modified hypothesized model and the first nested model (with the path representing the effect of T1 STS on T2 job burnout constrained to zero) was not significant. In contrast, the difference between the modified hypothesized model and the second nested model (with the path representing the effect of T1 job burnout on T2 STS constrained to zero) was significant. As in Study 1, the second nested model should be rejected. In sum, the results of the cross-lagged panel analysis suggested that the first nested model with the relationship between STS at T1 and job burnout at T2 constrained to zero should be accepted as a final model.

### Discussion

The results of Study 2 were consistent with the findings obtained in Study 1. Overall, a higher level of job burnout at T1 led to a higher level of STS at T2. In contrast, levels of STS at T1 did not predict job burnout at T2. Findings obtained among Polish healthcare and social workers providing services for civilians who had experienced traumatic events were similar to results obtained among the U.S. behavioral and mental healthcare providers working with the military personnel.

## General Discussion

The results of the two longitudinal studies provided new insights into the nature of the relationships between job burnout and STS. In particular, we found that job burnout may increase a risk of developing STS, but STS symptoms are unrelated to job burnout at follow-ups. The cross-lagged panel analyses of the U.S. and Polish samples of human services professionals who were indirectly exposed to traumatic events at work yielded consistent findings. The relationship between job burnout and STS seems to be unidirectional, with job burnout being a potential “gateway” outcome, enhancing the risk of developing STS.

Our investigation is one of the first to examine the directions in the associations of two core job-related outcomes affecting human services professionals working with clients exposed to traumatic events [3–5]. As such, it provides theorists and researchers with clues regarding the utility of existing models or frameworks, which account for both job burnout and STS symptoms [2,30]. The study findings reinforce arguments for including a unidirectional path from job burnout to STS into the theoretical models.

The findings are in line with the assumptions made in COR theory [31]. COR suggests that personal and environmental resources are depleted due to excessive expenditure to cope with a broad range of stressors (which may include work-related stressors) and their direct consequences (such as high levels of job burnout). This excessive expenditure leaves only few resources to cope with the further perpetual exposure to indirect trauma, making human services professionals susceptible to the development of STS symptoms. Previous studies found that job burnout relates to decline or low levels of various resources [43,44]. Our findings imply that loss spirals due to high levels of job burnout and limited resources remain critical to deal with indirect exposure to traumatic events. To break the loss spirals, new resources should be developed through prevention or treatment programs for human services professionals.

Unfortunately, the present research does not provide an insight about the type of resources, which could be main target of such prevention or treatment program. Previous research indicated that resources may include several organizational factors such as caseload size or diversity [30], which are difficult to change, but they may also include modifiable factors such as control beliefs or self-efficacy beliefs [21,30]. Recent meta-analyses indicated that such modifiable beliefs about one’s own ability to deal with stress and its consequences are linked to lower levels/a reduction of job burnout [12]. Therefore, self-efficacy and control belief may be included in resource-building prevention program for professionals at risk for burnout and subsequent STS. Future research may aim at conducting evaluations of effects of such programs on burnout (and consequently on STS).

Although the associations between job burnout and STS were similar in both studies, there were a few differences in parameters observed in the U.S. sample and the Polish sample. For example, the relationship between work experience and job burnout at T1 in the Polish sample was positive but negligible; in contrast, in the U.S. sample this association was significant and negative. Moreover, mean levels of job burnout and STS were higher in the Polish sample than in the U.S. sample. Unfortunately, it is not possible to elucidate whether the differences were due to cultural factors, differences in occupations of the samples, or the type of vicarious exposure (civilian vs military trauma). However, our findings are in line with previous research which reported higher levels of distress or ill health in Polish samples, compared with Western European or U.S. samples. For instance, Polish nurses had higher job burnout than Dutch nurses [45], higher stress and lower life satisfaction were found in a sample from general population drawn from Poland compared to a German sample [46], higher anxiety and depression were found in Polish college students compared to the students from the U.S. [47], and being Polish was a predictor for stronger PTSD among firefighters compared to being Czech, Italian, German, Spanish, Swedish, or Turkish [48]. Future research needs to clarify the sources of such differences, such as cultural factors, organizational factors, occupation-specific tasks and resources.

Furthermore, the differences in the findings obtained in the samples from Poland and the U.S. may be due to measurement issues. The test of measurement invariance of the STSS revealed that the factor structure of the STSS was different between the U.S. sample and the Polish sample. Again, the present study provides a limited insight into the cause of such differences. Differences in occupation, types of indirect exposure to trauma, availability of support systems for professionals, and their training could contribute to divergence of the factor structure of STSS in Poland’s and the U.S. samples. It is outside of the scope of the present research to distinguish the effects of these factors on the factor structure in the two samples. Future research should address this issue more comprehensively.

Although our data are suggestive of a causal path from job burnout to STS, correlational studies provide very limited arguments for causation. Although ethical concerns prohibit us from conducting experimental manipulations of either of the core constructs, larger scale natural experiments could be conducted to strengthen the conclusions. This study relied on the use of self-report measures, which, although relatively easy to obtain, are subject to a host of concerns. Clarke et al. [49] echoed such a concern regarding measures of depression in critiquing their randomized trial. Behavioral measures, other-ratings (including diagnostic interviews), and personnel records offer opportunities for more veridical measurement strategies and minimize artifacts such as mono-method bias. Finally, although our sample was somewhat diverse, the generalization across cultures and occupations would be premature.

In conclusion, our investigation showed that job burnout led to an increased frequency of STS symptoms at 6-month follow-up, but the levels of STS symptoms were not predictive of job burnout levels. The findings are robust, as cross-lagged panel analyses yielded similar results in two samples recruited in two different cultures, among workers performing various types of human services professions, and in the context of indirect exposure to military and civilian trauma. Our findings advance the knowledge of the process involving these two outcomes with the implications to theories explaining the effects of indirect traumatization as well as the prevention and may inform treatment programs dedicated to human services professionals dealing with traumatized clients.

## Supporting Information

S1 Table Correlation Matrix among the OLBI Items and the STSS Items in the U.S. Sample at Time 1. OLBI = Oldenburg Burnout Inventory; STSS = Secondary Traumatic Stress Scale. *p < .05; **p < .01; ***p < .001.
(PDF)

S2 Table Correlation Matrix among the OLBI Items and the STSS Items in the Polish Sample at Time 1. OLBI = Oldenburg Burnout Inventory; STSS = Secondary Traumatic Stress Scale. *p < .05; **p < .01; ***p < .001.
(PDF)
