# Supplementary material for: What Comes First, Job Burnout or Secondary Traumatic Stress? Findings from Two Longitudinal Studies from the U.S. and Poland
Source: PLoS One. 2015 Aug 25;10(8):e0136730. doi: 10.1371/journal.pone.0136730 (PMC4549333; doi:10.1371/journal.pone.0136730)
Supplement: S1 Table — OLBI = Oldenburg Burnout Inventory; STSS = Secondary Traumatic Stress Scale. *p < .05; **p < .01; ***p < .001. (PDF) [file pone.0136730.s001.pdf]

S1 Table. Correlation Matrix among the OLBI Items and the STSS Items in the U.S. Sample at Time 1

	STSS 1	STSS 2	STSS 3	STSS 4	STSS 5	STSS 6	STSS 7	STSS 8	STSS 9	STSS 10	STSS 11	STSS 12	STSS 13	STSS 14	STSS 15	STSS 16	STSS 17
**OLBI 1**	.32***	.23***	.07	.15**	.33***	.26***	.28***	.25***	.22***	.12*	.23***	.23***	.18**	.32***	.35***	.28***	.13*
**OLBI 2**	.21***	.23***	.06	.16**	.26***	.15*	.16**	.16**	.19***	.22***	.20***	.11	.18**	.27***	.26***	.19***	.15*
**OLBI 3**	.37***	.26***	.13*	.25***	.40***	.30***	.34***	.28***	.31***	.21***	.28***	.33***	.24***	.33***	.36***	.38***	.26***
**OLBI 4**	.42***	.21***	.21***	.33***	.38***	.34***	.37***	.37***	.40***	.35***	.37***	.35***	.35***	.38***	.40***	.32***	.22***
**OLBI 5**	.29***	.19***	.16**	.22***	.33***	.30***	.31***	.34***	.32***	.23***	.28***	.33***	.28***	.33***	.35***	.35***	.22***
**OLBI 6**	.36***	.24***	.14*	.16**	.28***	.24***	.30***	.20***	.25***	.12*	.24***	.27***	.16**	.28***	.35***	.29***	.24***
**OLBI 7**	.26***	.16**	.05	.15*	.30***	.18**	.29***	.21***	.27***	.14*	.21***	.25***	.12*	.27***	.33***	.19***	.17**
**OLBI 8**	.43***	.35***	.23***	.28***	.35***	.31***	.39***	.34***	.33***	.32***	.40***	.37***	.29***	.41***	.47***	.35***	.26***
**OLBI 9**	.29***	.16**	.09	.16**	.24***	.16**	.25***	.20***	.21***	.18**	.26***	.20***	.18**	.22***	.34***	.24***	.16**
**OLBI 10**	.33***	.18**	.16**	.30***	.31***	.26***	.34***	.28***	.39***	.29***	.28***	.35***	.26***	.20***	.34***	.26***	.21***
**OLBI 11**	.31***	.34***	.21***	.21***	.34***	.32***	.36***	.29***	.26***	.23***	.33***	.31***	.25***	.40***	.34***	.39***	.26***
**OLBI 12**	.30***	.25***	.07	.25***	.34***	.23***	.37***	.28***	.29***	.32***	.34***	.29***	.22***	.30***	.34***	.25***	.23***
**OLBI 13**	.14*	.11	.05	.09	.17**	.10	.15**	.14*	.12*	.06	.13*	.17**	.08	.23***	.23***	.14*	.14*
**OLBI 14**	.21***	.20***	.07	.09	.14*	.09	.21***	.25***	.26***	.12*	.27***	.21***	.14*	.12*	.24***	.25***	.22***
**OLBI 15**	.32***	.21***	.09	.23***	.36***	.28***	.34***	.29***	.31***	.15**	.25***	.31***	.21***	.28***	.39***	.31***	.24***
**OLBI 16**	.33***	.27***	.14*	.21***	.34***	.29***	.28***	.26***	.25***	.21***	.25***	.25***	.20***	.24***	.38***	.26***	.18**

Note. OLBI = Oldenburg Burnout Inventory; STSS = Secondary Traumatic Stress Scale. *$p < .05$; **$p < .01$; ***$p < .001$.
