# Supplementary material for: What Comes First, Job Burnout or Secondary Traumatic Stress? Findings from Two Longitudinal Studies from the U.S. and Poland
Source: PLoS One. 2015 Aug 25;10(8):e0136730. doi: 10.1371/journal.pone.0136730 (PMC4549333; doi:10.1371/journal.pone.0136730)
Supplement: S2 Table — OLBI = Oldenburg Burnout Inventory; STSS = Secondary Traumatic Stress Scale. *p < .05; **p < .01; ***p < .001. (PDF) [file pone.0136730.s002.pdf]

S2 Table. Correlation Matrix among the OLBI Items and the STSS Items in the Polish Sample at Time 1

	STSS 1	STSS 2	STSS 3	STSS 4	STSS 5	STSS 6	STSS 7	STSS 8	STSS 9	STSS 10	STSS 11	STSS 12	STSS 13	STSS 14	STSS 15	STSS 16	STSS 17
**OLBI 1**	.14*	.00	.10	.19***	.38***	.30***	.32***	.22***	.33***	.04	.23***	.31***	.21***	.26***	.29***	.27***	.19***
**OLBI 2**	.06	.13*	.13*	.22***	.34***	.27***	.32***	.28***	.29***	.22***	.29***	.26***	.28***	.24***	.28***	.21***	.08
**OLBI 3**	.09	.10	.23***	.35***	.49***	.42***	.38***	.41***	.43***	.26***	.35***	.38***	.29***	.31***	.38***	.32***	.22***
**OLBI 4**	-.01	.18**	.31***	.46***	.38***	.44***	.28***	.43***	.43***	.35***	.51***	.42***	.39***	.37***	.38***	.32***	.26***
**OLBI 5**	-.05	.10	.21***	.39***	.37***	.42***	.25***	.38***	.38***	.25***	.44***	.41***	.33***	.38***	.36***	.37***	.25***
**OLBI 6**	.12*	.02	.15*	.16**	.30***	.25***	.21***	.15**	.23***	.06	.19***	.27***	.17**	.18**	.28***	.19***	.23***
**OLBI 7**	.11*	-.01	.05	.14*	.22***	.24***	.24***	.21***	.25***	.14*	.20***	.31***	.24***	.22***	.25***	.20***	.16**
**OLBI 8**	-.03	.17**	.33***	.41***	.48***	.48***	.33***	.47***	.46***	.32***	.51***	.40***	.39***	.32***	.45***	.35***	.26***
**OLBI 9**	.15**	.11	.13*	.15**	.27***	.21***	.23***	.24***	.21***	.14*	.23***	.24***	.18**	.24***	.32***	.17**	.14*
**OLBI 10**	-.05	.17**	.26***	.39***	.45***	.46***	.36***	.40***	.46***	.27***	.37***	.39***	.35***	.30***	.30***	.32***	.20***
**OLBI 11**	.19***	.04	.09	.17**	.29***	.29***	.35***	.30***	.27***	.17**	.24***	.30***	.25***	.30***	.33***	.28***	.23***
**OLBI 12**	.05	.07	.22***	.24***	.38***	.36***	.29***	.34***	.38***	.27***	.37***	.34***	.25***	.24***	.36***	.26***	.20***
**OLBI 13**	-.14*	.06	.14*	.10	.11*	.12*	.07	.14*	.13*	.04	.17**	.18***	.12*	.17**	.10	.02	.05
**OLBI 14**	-.01	.09	.12*	.24***	.28***	.29***	.17**	.22***	.31***	.16**	.30***	.28***	.16**	.25***	.14*	.21***	.17**
**OLBI 15**	.05	.01	.07	.17**	.28***	.23***	.32***	.22***	.27***	.15**	.22***	.25***	.20***	.19***	.21***	.15*	.21***
**OLBI 16**	.10	-.03	.09	.16**	.34***	.33***	.37***	.35***	.43***	.13*	.32***	.39***	.17**	.26***	.32***	.28***	.20***

Note. OLBI = Oldenburg Burnout Inventory; STSS = Secondary Traumatic Stress Scale. *$p < .05$; **$p < .01$; ***$p < .001$.
